# Low-dose naltrexone for the treatment of fibromyalgia: protocol for a double-blind, randomized, placebo-controlled trial

**DOI:** 10.1186/s13063-021-05776-7

**Published:** 2021-11-15

**Authors:** Karin Due Bruun, Kirstine Amris, Henrik Bjarke Vaegter, Morten Rune Blichfeldt-Eckhardt, Anders Holsgaard-Larsen, Robin Christensen, Palle Toft

**Affiliations:** 1 Pain Research Group, Pain Center, Odense University Hospital, Heden 7-9, Indgang 20, DK – 5000 Odense C, Denmark; 2 Department of Clinical Research, Faculty of Health Sciences, University of Southern Denmark, Odense, Denmark; 3 The Parker Institute, Bispebjerg and Frederiksberg Hospital, Copenhagen, Denmark; 4 Department of Rheumatology, Bispebjerg and Frederiksberg Hospital, Frederiksberg, Denmark; 5 Department of Orthopedics and Traumatology, Odense University Hospital, Odense, Denmark; 6 Department of Anesthesiology and Intensive Care, Odense University Hospital, Odense, Denmark

**Keywords:** Fibromyalgia, Pain, Low dose naltrexone, LDN, RCT

## Abstract

**Background:**

Low-dose naltrexone (LDN) is used widely as an off-label treatment for pain despite limited evidence for its effectiveness. A few small trials with a high risk of bias have investigated the effect of LDN on pain associated with fibromyalgia in women, but larger and more methodologically robust studies are needed. The primary aim of this randomized controlled trial is to investigate if 12 weeks of LDN treatment is superior to placebo in reducing the average pain intensity during the last 7 days in women with fibromyalgia.

**Methods:**

A single-center, permuted block randomized, double-blind, placebo-controlled, parallel-group trial will be performed in Denmark. Randomization comprises 100 women aged 18–64 years diagnosed with fibromyalgia who will be treated with either LDN or placebo for 12 weeks including a 4-week titration phase. The primary outcome is change in average pain intensity (during the last 7 days) from baseline to 12 weeks. Secondary outcomes are other fibromyalgia-related symptoms, i.e., tenderness, fatigue, sleep disturbance, stiffness, memory problems, depression, anxiety and measures of global assessment, physical function, impact of fibromyalgia, pain distribution, and health-related quality of life. Intention-to-treat analysis will be performed, and the number of responders with a more than 15%, 30%, and 50% improvement of pain after 12 weeks will be calculated for the LDN and placebo groups. Exploratory outcomes include measures of pain sensitivity, muscle performance, and biomarkers.

**Discussion:**

This study will contribute with high-level evidence on the efficacy of low-dose naltrexone for the treatment of pain in women with fibromyalgia. Secondary outcomes include both disease-specific and generic components investigating whether LDN influences other symptoms than pain. Explorative outcomes are included to provide greater insight into the mechanism of action of LDN and possibly a better understanding of the underlying pathology in fibromyalgia.

**Trial registration:**

EudraCT 2019-000702-30. Registered on 12 July 2019. ClinicalTrials.gov NCT04270877. Registered on 17 February 2020

## Administrative information

Note: the numbers in curly brackets in this protocol refer to SPIRIT checklist item numbers. The order of the items has been modified to group similar items (see http://www.equator-network.org/reporting-guidelines/spirit-2013-statement-defining-standard-protocol-items-for-clinical-trials/).

**Table:** 

Title {1}	Low dose naltrexone for the treatment of fibromyalgia: Protocol for a double-blind, randomized, placebo-controlled trial
Trial registration {2a and 2b}.	EudraCT-number.: 2019-000702-30 ClinicalTrials.gov Identifier: NCT04270877
Protocol version {3}	Version 5.0 (25.09.2020). Amendment: Version 5.1 (27.07.2021)
Funding {4}	The Danish Rheumatism Association has granted 175.000 DKK for operating expenses. Odense University Hospital’s PhD fund for operation expenses has granted 100.000 DKK towards operating expenses and the study nurse’s salary. Aase and Ejnar Danielsens fund has granted 100.000 DKK towards operating expenses and the study nurse’s salary. The Parker Institute at Bispebjerg and Frederiksberg Hospital is supported by a core grant from the Oak Foundation (OCAY-18-774-OFIL). The work of the Primary Investigator (KDB) is covered by a PhD salary.
Author details {5a}	Karin Due Bruun*^1,2,3^ karin.due.bruun@rsyd.dk Kirstine Amris^3,4^ kirstine.amris@regionh.dk Henrik Bjarke Vaegter^1,2^ hbv@rsyd.dk Morten Rune Blichfeldt-Eckhardt^1,2^ mr.be@rsyd.dk Anders Holsgaard-Larsen^2,4^ AHLarsen@health.sdu.dk Robin Christensen^2,3^ robin.christensen@regionh.dk Palle Toft^2,6^ palle.toft@rsyd.dk ^1^ Pain Research Group, Pain Center, Odense University Hospital, Odense, Denmark ^2^ Department of Clinical Research, Faculty of Health Sciences, University of Southern Denmark, Odense, Denmark ^3^ The Parker Institute, Bispebjerg and Frederiksberg Hospital, Copenhagen, Denmark 4 Department of Rheumatology, Bispebjerg and Frederiksberg Hospital, Copenhagen, Denmark ^5^ Department of Orthopedics and Traumatology, Odense University Hospital, Odense, Denmark ^6^ Department of Anesthesiology and Intensive Care, Odense University Hospital, Odense, Denmark
Name and contact information for the trial sponsor {5b}	Palle Toft, Professor, MD, PhD, Dr. Med. Department of Anesthesiology and Intensive Care Odense University Hospital Sdr. Boulevard 29 5000 Odense C Email: palle.toft@rsyd.dk
Role of sponsor {5c}	The study is investigator initiated.

## Introduction

### Background and rationale {6a}

Low-dose naltrexone (LDN) has been used as an off-label treatment for pain and inflammation in multiple sclerosis, Crohn’s disease, and fibromyalgia (FM) for several years [1]. Naltrexone (NLX) is marketed as an additional therapy for the prevention of relapse in patients with previous abuse of opioids or alcohol [2]. While it is primarily known as an opioid receptor antagonist [3], NLX also attenuates dopaminergic transmission in mesolimbic pathways, thereby reducing cravings after substance abuse [4]. NLX has a similar biochemical structure to Naloxone but a higher oral bioavailability and a longer half-life [5], and it is well known that NLX can have a paradoxical analgesic effect when used in low doses of 1–6 mg [6].

The proposed mechanisms of action of LDN on pain are  1) opioid antagonism, which leads to a feedback-mediated increased expression of opioid receptors in the central nervous system (CNS) [7, 8] with a possible improvement of the endorphin system and 2) an anti-inflammatory effect, mediated through inhibition of Toll-like receptor 4 (TLR4) on astrocytes and microglia cells, thereby possibly inhibiting the pro-inflammatory cytokine cascade thought to be involved in the development and maintenance of chronic pain [9, 10].

The evidence for an analgesic effect of LDN is sparse, however. Several case reports exist [11–13], but only three small clinical trials have been published. The first trial was a single-blind pilot study with participation of 10 women with FM [14]. The subjects received placebo for 2 weeks, followed by an 8-week treatment with LDN 4.5 mg. Quantitative sensory testing showed improved pressure pain and heat pain thresholds during treatment with LDN compared to placebo. The same research team conducted a double-blind, placebo-controlled, randomized (cross-over) trial (RCT) [15], where 31 women with FM were randomized to receive either 4-week treatment with placebo followed by 12-week treatment with LDN 4.5 mg or 12-week treatment with LDN 4.5 mg followed by 4-week treatment with placebo. Both studies found LDN to be significantly better than placebo in reducing pain. The third and most recent study was a single-blind non-controlled pilot study with participation of 8 women with FM [16]. The participants were told they could receive placebo at any time during the 8-week intervention, but all patients received active treatment (LDN 4.5 mg) throughout the trial. Significant reductions from baseline were seen in 17 out of 63 pro-inflammatory cytokines, supporting the hypothesis of an anti-inflammatory effect of LDN. The two pilot studies represent important pioneer work, but do not provide high-level evidence because of lack of power and single-blind or non-controlled study designs with a high risk of bias. In the cross-over trial, the method is more robust with both randomization and double-blinding. However, the study also has some weaknesses. Although showing promising results, it is unclear if the study was sufficiently powered and the decision to exclude a washout period between the interventions increases the risk of bias.

LDN has been shown to be a safe treatment [17] and a low-cost alternative to traditional therapies, but larger RCTs are needed to confirm its potential efficacy in reducing pain in patients diagnosed with fibromyalgia. Previous trials investigating the effect of LDN on pain have used one daily dose of 4.5 mg. However, higher doses might be more beneficial for some patients. Our study group previously conducted a dose-response study testing doses in the range of 0.75–6 mg [18]. We found the effective dose in 50% (ED50) to be 3.88 and the effective dose in 95% (ED95) to be 5.40 mg. We concluded that 4.5 mg would be a relevant test dose as it lies in the range between ED50 and ED95. However, doses closer to ED95 would be expected to be even more efficacious. As we found no problems with tolerability using doses in the range from 4.5 to 6 mg, we decided to test 6 mg against placebo in this RCT.

## Objectives {7}

The primary objective is to investigate if 12 weeks’ treatment with 6 mg LDN is superior to placebo in reducing the average pain intensity (during the last 7 days) in women with fibromyalgia. Secondary objectives include evaluating the clinical effect on 21 secondary outcomes covering core symptoms, daily functioning, impact of FM, quality of life, global impression of change, and responder indices. Finally, we will explore effects on pressure pain thresholds, temporal summation of pain, conditioned pain modulation, physical fitness, muscle exhaustion, and blood levels of pro-inflammatory cytokines.

## Trial design {8}

The study is designed as a single-center, permuted block randomized, double-blind, placebo-controlled, parallel-group trial. Randomization comprises a parallel randomized (1:1) allocation of 100 women aged 18–64 years diagnosed with fibromyalgia, treated with either LDN or placebo for 12 weeks including a 4-week titration phase (from baseline to week 4).

## Methods: participants, interventions, and outcomes

### Study setting {9}

The study is a single-center study that is conducted at a public university hospital in Southern Denmark (SMERTECENTER SYD, Heden 7-9, 5000 Odense C). The setting is a tertiary pain rehabilitation center.

### Eligibility criteria {10}

Inclusion criteria:

- Women aged 18–64 years
- Can understand and write Danish
- Fulfill the American College of Rheumatology 1990 criteria for FM [19]
- A minimum score of 4 for self-reported average pain during the last 7 days on a 0–10 numeric rating scale (NRS) at baseline
- Women of child-bearing age must use safe contraception (spiral, birth control pills, contraceptive patch, contraceptive vaginal ring, or gestagen injections) for 3 weeks before and 1 week after the trial. If a participant’s usual lifestyle includes sexual abstinence, contraception is not required, but the participant must give oral informed consent that they will remain sexually abstinent during the trial

Exclusion criteria:

- Known allergy to naltrexone hydrochloride
- Pregnancy or breastfeeding; a negative pregnancy test must be available at baseline for all women of fertile age
- Use of opioids or NSAIDs up to 4 weeks before inclusion in the trial
- Known abuse of alcohol or other substances
- Known inflammatory rheumatic disease
- Known demyelinating disease
- Known active cancer
- Liver dysfunction (alanine aminotransferase (ALAT) must not be elevated more than 2-fold over the highest reference level)
- Kidney dysfunction (glomerular filtration rate (GFR) must not be below 59 mL/min)
- Psychotic disease
- History of a suicide attempt
- Suicide ideation—evaluated using Patient Health Questionnaire—9 items (PHQ-9) [20]; item 9 must be answered “never”

### Who will take informed consent? {26a}

Potential participants recruited from the pain center will receive written information about the trial from their nurse or physician. For potential participants recruited via advertising, written information is sent by e-mail. All potential participants receive a telephone call from the primary investigator (PI) (author KDB), who gives oral information about the trial. It is emphasized that participation is voluntary and that consent can be withdrawn at any time. A minimum of 24 h is given for reflection. The PI obtains the informed consent before inclusion.

### Additional consent provisions for collection and use of participant data and biological specimens {26b}

Informed consent to use blood from the biobank to perform analyses for other research purposes is obtained from all participants.

## Interventions

### Explanation for the choice of comparators {6b}

No comparators are used other than the identically appearing placebo control.

### Intervention description {11a}

After inclusion, the participants will be randomized using a computerized algorithm to receive either placebo or LDN for 12 weeks. The participants’ dose will be titrated up to 6 mg following a dose-escalation scheme: an initial dosage of 1.5 mg daily, escalated every seventh day by 1.5 mg up to 6 mg at week 4. Dose escalation will be based on safety and tolerability, and if dose escalation is not feasible, delayed increments are allowed. For the surveillance of harms, both active and passive methods will be used. The participants will be encouraged to report adverse events spontaneously and will be asked about the occurrence of specific common side effects by administering a questionnaire. For the graduation of the severity of harms, the Common Terminology Criteria for Adverse Events (CTCAE) version 5.0 will be used. If the participant reports harms categorized as grade 2 or higher, they will be advised by the primary investigator to lower the dose. If harms are categorized as grade 1, the decision about dosing will be made individually in agreement between the primary investigator and the participant. After the end of week 4, the dose will be fixed for the rest of the trial, as the highest dose tolerated at this time point. The trial medicine is taken once daily in the evening, between 7 pm and 11 pm.

### Criteria for discontinuing or modifying allocated interventions {11b}

The participants will be maintained at 6 mg (or the highest tolerated dose level established after the end of week 4) for the last 8 weeks of the treatment period. It is not allowed to increase the dose during the last 8 weeks. If problems with tolerability should arise during the last 8 weeks of treatment, it is allowed to lower the dose or discontinue treatment. Participants who alter the dose during the last 8 weeks of the trial will be considered not adherent to the protocol, but will be included in the intention-to-treat analysis.

### Strategies to improve adherence to interventions {11c}

Participants will receive a daily short text message (SMS) reminding them to take their trial medication. At all visits, empty medicine cans are returned, and non-ingested tablets are counted.

### Relevant concomitant care permitted or prohibited during the trial {11d}

The use of opioids, NSAIDs, and other drugs with an anti-inflammatory effect is prohibited during the trial. Participants can continue their usual care during the trial, but their pain medication has to be stable. The participants are not allowed to receive any new pain medication during the trial. Changes in concomitant medication are monitored at every visit via the patient’s shared electronic medication record.

### Provisions for post-trial care {30}

In the case of adverse events or adverse reactions, the PI will follow up on the participants until the symptoms have ceased or are stable. The participants are covered by the governmental patient insurance, which covers all patients in the Danish health care system.

### Outcomes {12}

As previous studies have shown significant reductions in pain intensity in women with FM treated with LDN 4.5 mg for 8–12 weeks, we have chosen the primary outcome to be change in average pain intensity (during the last 7 days) from baseline to 12 weeks of intervention. The 21 secondary outcome measures were chosen among measures that could potentially support a clinical effectiveness claim as recommended by the Outcome Measures in Rheumatology Clinical Trials (OMERACT) guidelines [21]. All patient-reported outcomes will be collected at baseline and after 4, 8, and 12 weeks.

#### Primary outcome measure

Change from baseline to 12 weeks of treatment in average pain intensity during the last 7 days on an 11-point rating scale (ranging from 0 = “no pain” to 10 = “unbearable pain”) using the first item from the symptom part of the Fibromyalgia Impact Questionnaire Revised (FIQR) [22].

#### Secondary outcome measures

The secondary outcomes include 21 supportive measures that will be collected, analyzed, and reported in the primary manuscript.

For the following secondary outcomes, the between-group change at baseline compared to 4, 8, and 12 weeks of treatment will be assessed:

1. Global assessment: assessed by Patient Global Impression of Change on a 1–7 Verbal Rating Scale
2. Impact of fibromyalgia: assessed by the FIQR total score [22]
3. Pain distribution: assessed by the Widespread Pain Index (WPI) from the 2016 diagnostic criteria for fibromyalgia [23]
4. Level of pain (assessment of pain intensity trajectory): assessed by the FIQR “level of pain” question
5. Level of tenderness: assessed subjectively by the FIQR “level of tenderness to touch” question and objectively by measurement of pressure pain threshold, using a handheld algometer. Algometry is performed only at baseline and after 12 weeks of treatment
6. Level of fatigue: assessed by the FIQR “level of energy” question
7. Level of sleep disturbance: assessed by the FIQR “quality of sleep” question
8. Level of depression: assessed by the FIQR “level of depression” question
9. Level of anxiety: assessed by the FIQR “level of anxiety” question
10. Level of cognition: assessed by the FIQR “level of memory problems” question
11. Level of stiffness: assessed by the FIQR “level of stiffness” question
12. Level of physical function: assessed by the physical function domain of FIQR
13. Health-related quality of life - mobility: assessed by the EQ-5D-5L mobility domain
14. Health-related quality of life - self-care: assessed by the EQ-5D-5L self-care domain
15. Health-related quality of life - usual activities: assessed by the EQ-5D-5L usual activities domain
16. Health-related quality of life - pain/discomfort: assessed by the EQ-5D-5L pain/discomfort domain
17. Health-related quality of life - anxiety/depression: assessed by the EQ-5D-5L anxiety/depression domain
18. Health-related quality of life - global: assessed by the EQ-5D Visual Analogue Scale (EQ-VAS)

Responder indices are calculated:

19. Number of responders with a more than 15% improvement of the primary outcome
20. Number of responders with a more than 30% improvement of the primary outcome
21. Number of responders with a more than 50% improvement of the primary outcome

#### Exploratory secondary outcomes (not to be reported in the primary manuscript)

The following exploratory outcomes will be investigated and reported in secondary publications. For the patient-reported outcome (variation in pain), the between-group change between baseline and after 8 and 12 weeks of treatment is measured. For all the protocol-specific procedures, the between-group change between baseline and after 12 weeks of treatment is measured.

- Variation in pain: assessed using a diary of daily average pain rated on an 11-point rating scale during 7 days before visits. The highest score minus the lowest score characterizes the variation in pain
- Muscle exhaustion: measured by an isometric muscle exhaustion test of the deltoid muscle
- Physical fitness: measured by the 30-s chair stand test
- Pain sensitivity: measured by computerized pressure cuff algometry (CPA)
- Inhibition of pain: measured by CPA using conditioned pain modulation (CPM)
- Augmentation of pain: measured by CPA using temporal summation of pain (TSP)

Blood for a biobank will be collected before baseline and immediately after 12 weeks of treatment for later analysis of pro- and anti-inflammatory cytokines. A separate protocol will be made to determine which cytokines will be investigated before the analyses are carried out.

### Participant timeline {13}

**Fig. 1:**
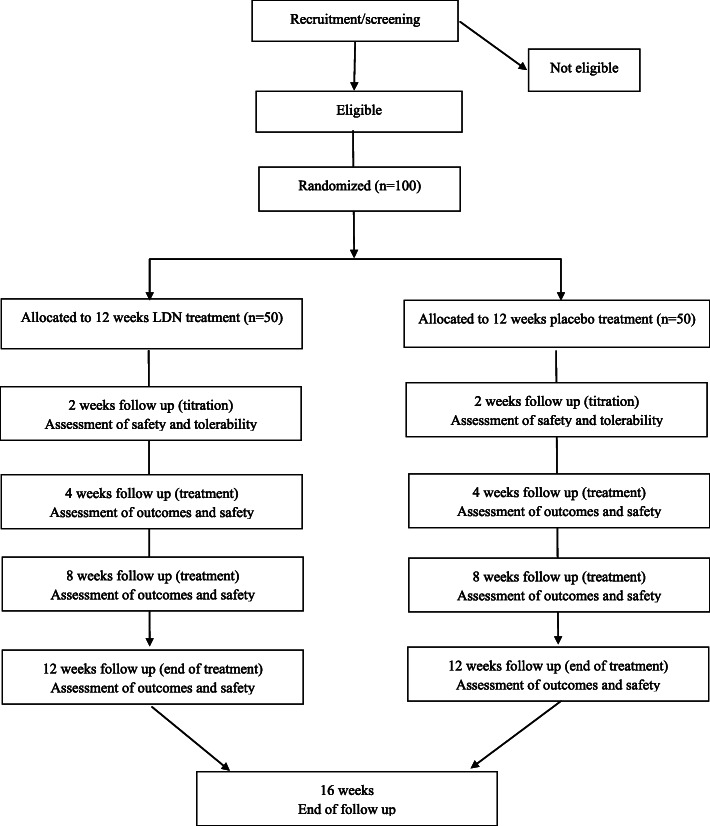
Overview of the participant flow

The participant flow is shown in Fig. 1. A time schedule for enrolment, interventions, and assessments is presented in Table 1.

**Table 1:** Schedule of enrolment, interventions, and assessments

	Study period						
	Enrolment	Allocation	Post-allocation				Follow-up
Week	***−4–0***	0	***2***^a^ ***(telephone)***	***4***^a^	***8***^**b**^	***12***^b^	***16***^b^
**Enrolment**							
**Informed consent**	X						
**Medication history**	X	X	X	X	X	X	X
**Demographic data**	X						
**Eligibility screen**	X						
**Allocation**		X					
**Interventions**							
**Low-dose naltrexone**							
**Placebo**							
**Assessments**							
**Vital tests**: blood pressure, weight, height		X				X	X
**Safety tests:** ALAT, creatinine, GFR, thrombocyte count, bilirubin. ECG	X					X	
**hCRP**		X					
**Blood for biobank**		X				X	
**PROMs**							
PHQ-9	X						
GAD-7	X						
FIQR		X		X	X	X	X
PGI-C				X	X	X	X
EQ-5D		X		X	X	X	X
EQ-VAS		X		X	X	X	X
**Pain sensitivity**							
Handheld algometry		X				X	
Computerized cuff algometry		X				X	
**Muscle tests**							
Isometric muscle exhaustion of deltoid		X				X	
30-s stand chair test		X				X	
**Compliance assessment**						X	
**Adverse events**		X	X	X	X	X	X

*ALAT* alanine aminotransferase, *GFR* glomerular filtration rate, *ECG* electrocardiogram, *PHQ-9* Patient Health Questionnaire – 9 items, *GAD-7* Generalized Anxiety Disorder – 7 items, *hCRP* high-sensitive C-reactive protein, *FIQR* Fibromyalgia Impact Questionnaire Revised, *PGI-C* Patient Global Impression of Change, *EQ-5D* EuroQol 5 dimensions, *EQ-VAS* EuroQol Visual Analogue Scale

^a^±2 days

^b^±7 days

### Sample size {14}

Using values from our previous dose-response study [18], we determined that self-reported pain on a 0–10 NRS at baseline had a mean of 6.7 in the target population, with a standard deviation (*SD*) of 1.5 NRS points. According to the Initiative on Methods, Measurement, and Pain Assessment in Clinical Trials (IMMPACT) guidelines [24], a minimal clinical important difference (MCID) is defined as a 15% decrease in pain [24], corresponding to a reduction of 1.0 NRS points in the present population. Using an MCID of 1.0 NRS, an *SD* of 1.5, a statistical power of at least 80%, and a statistical significance level of 0.05, a total of 74 patients are required, i.e., 37 patients in each group. Expecting some attrition and drop-out during the 12-week trial period, we decided to include 100 patients (with approximately 50 patients in each group), corresponding to a statistical power of more than 90% to detect a difference between groups in the ITT population.

If the intended sample size is not reached at 30 months after recruitment has started, the inclusion of patients will stop at 74 patients, which will ensure a power of 80%.

### Recruitment {15}

Participants are recruited from a pain center at a public university hospital and through advertisement in relevant written and social/Web-based media. For ethical reasons, patients in active treatment at the pain center will not be recruited, but only patients who have completed treatment and signed up for participation in future medical trials or waiting list patients. To secure a broad representation of FM severity to the study population, recruitment through advertisement will be equally favored.

## Assignment of interventions: allocation

### Sequence generation {16a}

A computerized algorithm will be generated for randomization by preparing a list of 100 sequential numbers to active intervention or placebo intervention; randomization will be based on permuted blocks of 2–6 individuals. No stratifications are applied to the randomization, and both investigators and outcome assessors are blinded regarding the permuted blocking strategy.

### Concealment mechanism {16b}

A data manager, with no clinical involvement in the trial, prepares the randomization sequence. The allocation is concealed in a password-protected computer file that is only accessible by the data manager. The randomization list is sent to the hospital pharmacy, who labels the medicine with blinding codes according to this list. The medicine is then shipped to the place of the trial. Unblinding will not take place before primary analysis of the data has taken place. In case unblinding of a single participant is necessary during the trial, individual allocations will also be held in sealed, opaque, consecutively numbered envelopes.

### Implementation {16c}

The PI enrolls all participants. After signing the informed consent form, each participant is allocated a sequential number that randomizes them to one of the two groups.

## Assignment of interventions: blinding

### Who will be blinded {17a}

The study is triple-blind as participating patients, investigators, and outcome assessors (and statistical analysts) are blinded to the allocation. The active medicine and placebo tablets will look identical and will be blinded in similar cans and labeled with blinding codes.

### Procedure for unblinding if needed {17b}

In the case of a suspected unexpected serious adverse reaction (SUSAR), the participant will be unblinded by the sponsor before reporting to the Danish Medicines Agency, but the PI will remain blinded. The PI will only be unblinded in the case of a medical emergency and only if the PI finds it necessary to ensure the safety of the subject. The PI can unblind a single subject by breaking the code-envelope for the subject’s code number.

## Data collection and management

### Plans for assessment and collection of outcomes {18a}

After allocation has taken place, the participants will complete questionnaires at the beginning of every visit via an electronic survey and before talking to the investigators. The Fibromyalgia Impact Questionnaire Revised [22] is a disease-specific instrument, while the EQ-5D-5L (which includes the EQ-VAS) [25] is a generic instrument. All are validated for use in clinical trials.

The level of tenderness is assessed at baseline and after 12 weeks of treatment using a handheld pressure algometer (Somedic Algometer, Hørby, Sweden). Assessment sites are the right quadriceps muscle 15 cm from the apex patella and the left trapezius muscle 10 cm from acromion (between acromion and C6/7). Each site is assessed three times. To avoid bias due to interrater variability, the same investigator will carry out all the procedures.

The exploratory outcome measures are assessed by an independent assessor at baseline and after 12 weeks of treatment. Standard operating procedures will be available, and the assessor will be trained in the procedures before and during the trial. The procedures are:

- Computer-controlled cuff algometry on lower legs in all participants to assess pressure pain threshold, pressure pain tolerance, temporal summation of pain, and conditioned pain modulation. Standardized assessment of experimental pressure pain sensitivity has shown good reliability and provides insights into the pathophysiological mechanisms involved in the pain condition.
- Muscular exhaustion: the participant completes an isometric muscle exhaustion task by maintaining 90° shoulder abduction (dominant arm) for as long as possible with the elbow extended and the hand pronated (hand facing downwards). Task failure (test position can no longer be maintained) defines the test duration. Surface electromyography (EMG) will be recorded from the anterior, middle, and posterior deltoid muscle at 3000 Hz during the entire test. The test has been shown to be feasible in women with fibromyalgia [26].
- Physical fitness is measured by the 30-s chair stand test, which has been shown to be reliable and feasible in women with fibromyalgia [27].

### Plans to promote participant retention and complete follow-up {18b}

The participants will receive a daily short text message (SMS) reminding them to take their trial medication. Participants who discontinue the treatment during the trial will be encouraged to complete all visits as scheduled.

### Data management {19}

The participants enter questionnaire data directly via a survey into the electronic Case Report File (eCRF) using REDCap electronic data capture tools. The EMG files are saved in a secured and logged Sharepoint. Results from the protocol-specific procedures will be collected in paper format and then entered into the eCRF. The assessors enter all other data directly into the eCRF during the visits. Data quality in the eCRF will be promoted using range checks for data values. Data will later be transferred to a statistical program for analyses. The data will be anonymized 5 years after the termination of the study.

### Confidentiality {27}

All data about potential and enrolled participants will be collected in a secure and logged database, in a secure and logged Sharepoint, or behind a double lock for data in paper format. Only anonymized data will be shared.

### Plans for collection, laboratory evaluation, and storage of biological specimens for genetic or molecular analysis in this trial/future use {33}

Blood for a research biobank will be collected before baseline and after 12 weeks of treatment. The purpose of the biobank is to be able to measure a possible change in pro- and anti-inflammatory cytokines in participants receiving active treatment compared to placebo. For this purpose, 2 × 0.5 ml serum and 2 × 0.5 ml plasma are collected before baseline and after 12 weeks of treatment. Any excess blood will be stored for 10 years. After 10 years, the blood will be destroyed. Informed consent to perform analyses for other research purposes is collected from all participants.

## Statistical methods

### Statistical methods for primary and secondary outcomes {20a}

The main analyses will be based on the intention-to-treat (ITT) population. This ITT principle asserts the effect of a treatment policy (that is, the planned treatment regimen) rather than the actual treatment given (i.e., it is independent of treatment adherence). Accordingly, participants allocated to a treatment group at baseline (*X*_LDN_ or *X*_Placebo_) will be followed up, assessed, and analyzed as members of that group, irrespective of their adherence to the planned course of treatment (i.e., independent of withdrawals and cross-over phenomena). By using mixed effects models (explained below), missing data after baseline will be handled indirectly; mixed effects models are valid assuming data are “Missing at Random” (MAR) [28].

All *P* values and 95% confidence intervals (*95% CI*) will be two-sided. We will not apply explicit adjustments for multiplicity; rather, we will analyze and interpret the 21 secondary outcomes in a prioritized order (e.g., “gatekeeping procedure” and/or the Hochberg sequential procedure). The analyses of the key secondary outcomes will be performed in sequence until one of the analyses fails to show the statistically significant difference, or until all analyses have been completed at a statistical significance level of 0.05 (i.e., *95% CI* does not overlap “the null”).

Unlike the Bonferroni correction/interpretation (directly adjusting the statistical significance threshold by the number of tests planned [say, *k*] → P* = 0.05/*k*), we will apply the Hochberg sequential procedure, where all the tests are performed and the resultant *P* values are ordered from largest to smallest on a list [29]. With our statistical significance level fixed at 5% and the largest observed if the *P* value is less than .05, then all the tests will be considered significant. Otherwise, if the next largest *P* value is less than 0.05/2 (.025), then all the tests except the one with the largest *P* value are considered significant. This process will be continued until all the comparisons made have been interpreted. This approach uses progressively more stringent statistical thresholds with the most stringent one being the Bonferroni threshold. This approach will achieve a greater power to detect true effect than the Bonferroni procedure [30].

Our primary (main) analyses will be based on the estimation of between-group differences in the continuous outcomes after 12 weeks for primary and secondary outcomes. Repeated measurements (*T* = 0, 4, 8, and 12 weeks from baseline) are used based on a linear mixed model where the treatment group is used as a fixed effect and participant ID as a random-effect parameter. All between-group differences will be adjusted for baseline level in order to reduce the random variation. The primary statistical model will consist of fixed effects and random effects. Fixed effects define the expected values of the observations, and random effects define the variance and covariances of the observations. In this study, participants will be randomly assigned to two treatment groups (*X*_LDN_ vs *X*_Placebo_), and observations are made at four time points for the primary outcome measure (baseline and 4, 8, and 12 weeks from baseline). Basically, there are two fixed-effect factors: group and time. Random effects result from variation between and within participants. We anticipate that measures on the same patient at different times are correlated, with measures taken closely together in time being more highly correlated than measures taken more apart in time. Observations on different participants will be assumed to be independent.

Secondarily, an analysis of the number of responders (dichotomous outcomes) in the two groups will be carried out using logistic regression analyses. A responder is defined as a participant who reports a more than 15%, 30%, or 50% decrease in pain after 12 weeks of treatment with LDN. For these dichotomous outcomes, logistic regression will be used to calculate the odds ratio (*OR*) with *95% CI* comparing the two groups. For subsequent ease of interpretation, the *OR* values will be converted into (relative) risk ratios and (absolute) risk differences. The pre-specified efficacy analyses will be based on the data for the full analysis set, the ITT population, which includes all participants assessed and randomized at baseline.

### Interim analyses {21b}

Not applicable as no interim analysis is made.

### Methods for additional analyses (e.g., subgroup analyses) {20b}

Not applicable as no subgroup analyses are made.

### Methods in analysis to handle protocol non-adherence and any statistical methods to handle missing data {20c}

Repeated measurements using mixed models will be based on the ITT population, including all randomized participants with available data at baseline. Missing data will be handled indirectly and statistically modeled using repeated-measures linear mixed models (see below). These models will be valid if data are missing at random (MAR): “Any systematic difference between the missing values and the observed values can be explained by differences in observed data” [28]. Contrasts between groups will be estimated based on repeated-measures analysis of covariance applied in mixed linear models (at 12 weeks from baseline). Thus, in the case of missing data during the 12-week trial, repeated-measures linear mixed models will adjust for that indirectly.

To confirm the robustness of the findings for the primary and key secondary outcomes, sensitivity analyses will be performed on the main analyses including the:

(i) “Complete Case” population, i.e., outcome data recorded both at baseline and after 12 weeks; a dataset potentially valid if data are missing completely at random (MCAR)
(ii) Non-responder imputation: use of single imputation where the baseline observation is carried forward; potentially valuable if data are not missing at random (NMAR)
(iii) “Per Protocol” population: defined as participants with at least 80% adherence to treatment

Robustness is a concept that refers to the sensitivity of the overall conclusions to various limitations of the data, assumptions, and analytic approaches to data analysis. Robustness implies that the treatment effect and primary conclusions of the FINAL trial are not substantially affected when analyses are carried out based on alternative assumptions or analytic approaches.

### Plans to give access to the full protocol, participant-level data, and statistical code {31c}

The full protocol and the statistical analysis plan (SAP) will be accessible at www.clinicaltrials.gov, identifier: NCT04270877.

## Oversight and monitoring

### Composition of the coordinating center and trial steering committee {5d}

Not applicable as it is a single-center study.

### Composition of the data monitoring committee, its role, and reporting structure {21a}

The Good Clinical Practice (GCP) unit at Odense University Hospital monitors the trial.

### Adverse event reporting and harms {22}

Data on adverse events (AEs) and adverse reactions (ARs) are collected at all visits. The participants will complete a questionnaire about the presence of known side effects and will be interviewed by the PI about any adverse events that occur during the trial. For the graduation of the severity of harms, the Common Terminology Criteria for Adverse Events (CTCAE) version 5.0 will be used. The PI assesses whether an AE is related to the trial medication using the Summary of Product Characteristics (SmPC) for Naltrexone 50 mg as a reference document. All AEs and ARs are described in detail and registered in the eCRF.

ALAT, bilirubin, creatinine, GFR, thrombocyte count, and electrocardiogram are assessed before and after the intervention. Urinary human chorionic gonadotropin is measured at baseline (week 0) and after 4, 8, and 12 weeks of treatment in all women of fertile age.

A serious adverse event (SAE) is any untoward medical occurrence or effect that at any dose results in death, is life-threatening, requires hospitalization or prolongation of existing hospitalization, results in persistent or significant disability or incapacity, or is a congenital anomaly or birth defect. All SAEs are reported by the PI to the sponsor within 24 h. Causality of an SAE will be determined according to the detailed guidance on the collection, verification, and presentation of adverse event/reaction reports arising from clinical trials on medicinal products for human use (CT-3) guidelines. If a serious adverse reaction (SAR) is assessed as unexpected according to the SmPC, the sponsor must unblind the subject before reporting it to the Danish Medicines Agency. The PI will remain unblinded. Under section 89 [2](i) of the Danish Medicines Act, the sponsor must immediately inform the Danish Medicines Agency if any SUSARs occur during the trial.

### Frequency and plans for auditing trial conduct {23}

Not applicable as no auditing.

### Plans for communicating important protocol amendments to relevant parties (e.g., trial participants, ethical committees) {25}

Any important protocol amendments will be reported to the Danish Medicines Agency, the local ethical committee, the monitor of the trial, participating investigators, trial participants, relevant trial registries, and the journal that has published the protocol.

## Dissemination plans {31a}

Information about the trial is published at ClinicalTrials.gov and the European Union Drug Regulating Authorities Clinical Trials Database (EUDRACT) before enrolment of the first patient. The protocol and study results will be published in international peer-reviewed journals. Both positive, negative, and inconclusive results will be published. After publication, the results from the trial will be disseminated to the trial participants via email and to the public via written and Internet media.

## Discussion

The traditional pharmacological treatment of chronic non-malignant pain (CNMP), which includes FM, aims at reducing facilitatory neurotransmitters (e.g., gabapentinoids) or increasing inhibitory neurotransmitters (e.g., serotonin-norepinephrine reuptake inhibitors) [31]. These treatments do not always result in satisfactory pain relief, however, and their use is often limited by side effects. Furthermore, traditional therapies do not necessarily offer relief from other key symptoms associated with CNMP/FM. The results of our previous dose-response study indicated that LDN has a positive influence on sleep disturbance, energy, and touch tenderness in women with FM [18]. This is in concordance with previous trials on efficacy [14, 15]. Thus, treatment with LDN might offer several advantages to existing treatments such as new targets of action, fewer side effects, and a relatively low cost.

Currently, LDN is widely used as an off-label treatment for CNMP including FM, but the evidence is based on case reports and a few small clinical trials. This will be the first high-quality trial of LDN with a sufficient sample size to investigate a clinically relevant change in pain in women with FM. In addition, the current randomized, placebo-controlled trial aims to provide high-quality evidence by reducing the risk of bias through blinding of participating patients, investigators, outcome assessors, and statistical analysts. Finally, the transparency of the applied methods and definitions of outcome measures will be ensured through public access to the current protocol paper and a priori registration at ClinicalTrials.gov.

This trial contains both pragmatic and exploratory elements. The study will be the first to explore the efficacy of 6 mg LDN in women with fibromyalgia. However, for pragmatic reasons, a titration phase allows the testing of lower doses in case of problems with tolerability, aiming to assess the effectiveness of LDN on pain and other FM symptoms. Another pragmatic attitude is to secure a broad spectrum of FM severity recruiting participants through advertising and allowing for continued use of different kinds of usual care. The inclusion of exploratory outcomes aims to examine the mechanisms of action of LDN. If an effect of LDN on pain sensitivity, muscle fatigue, or biomarkers for CNS inflammation can be demonstrated in women with FM, this will not only expand our knowledge about mechanisms of action of LDN but might also contribute to a better understanding of underlying pathology in FM.

FM represents a well-defined subgroup of CNMP that is suitable for clinical trials. The disorder is characterized by chronic widespread pain and widespread hyperalgesia to mechanical stimulation [19] and is a classic example of a nociplastic pain disorder hypothesized to be caused primarily by disturbances in central pain regulatory mechanisms [32, 33]. Findings from trials in FM patients might therefore be extrapolated to other primary pain conditions with nociplastic pain features. As FM is diagnosed more frequently in women [34], recruitment of men with FM can be difficult. We have therefore chosen to include only women in order to ease recruitment and strengthen the internal validity of the results. This will have an impact on generalizability and external validity, and the final results must be reproduced later in a population including men.

## Trial status

Protocol version 5.1. Date: 27.07.2021 (dd.mm.yyyy)

Approval from authorities: 30.10.2019

Expected start of inclusion: 01.11.2020

Expected end of inclusion: 01.01.2023

Expected end of follow-up:01.06.2023

## Abbreviations

LDN: Low-dose naltrexone
FM: Fibromyalgia
NLX: Naltrexone
CNS: Central nervous system
TLR4: Toll-like receptor 4
RCT: Randomized controlled trial
NRS: Numeric rating scale
ALAT: Alanine aminotransferase
GFR: Glomerular filtration rate
PHQ-9: Patient Health Questionnaire – 9 items
PI: Primary investigator
SMS: Short text message
OMERACT: Outcome Measures in Rheumatology Clinical Trials
FIQR: Fibromyalgia Impact Questionnaire Revised
WPI: Widespread Pain Index
EQ-VAS: EQ-5D visual analog scale
CPA: Computerized pressure cuff algometry
CPM: Conditioned pain modulation
TSP: Temporal summation of pain
IMMPACT: The Initiative on Methods, Measurement, and Pain Assessment in Clinical Trials
MCID: Minimal clinical important difference
ITT: Intention-to-treat
SUSAR: Suspected unexpected serious adverse reaction
EMG: Surface electromyography
eCRF: Electronic Case Report File
*CI*: Confidence interval
*OR*: Odds ratio
MAR: Missing at random
MCAR: Missing completely at random
NMAR: Not missing at random
SAP: Statistical analysis plan
GCP: Good Clinical Practice
AE: Adverse event
AR: Adverse reaction
SmPC: Summary of Product Characteristics
SAE: Serious adverse event
CT-3: The detailed guidance on the collection, verification, and presentation of adverse event/reaction reports arising from clinical trials on medicinal products for human use
SAR: Serious adverse reaction
EUDRACT: European Union Drug Regulating Authorities Clinical Trials Database
CNMP: Chronic non-malignant pain
